# Inflammatory Cytokines During Cardiac Rehabilitation After Heart Surgery and Their Association to Postoperative Atrial Fibrillation

**DOI:** 10.1038/s41598-020-65581-1

**Published:** 2020-05-25

**Authors:** Vittorio Racca, Anna Torri, Paola Grati, Claudia Panzarino, Ivana Marventano, Marina Saresella, Paolo Castiglioni

**Affiliations:** 1 IRCCS Fondazione Don Carlo Gnocchi, Cardiology Rehabilitation Department, Milan, Italy; 2 IRCCS Fondazione Don Carlo Gnocchi, Milan – Italy, Milan, Italy; 3 IRCCS Fondazione Don Carlo Gnocchi, Laboratory of Molecular Medicine and Biotechnology, Milan, Italy

**Keywords:** Diagnostic markers, Atrial fibrillation

## Abstract

Inflammation is associated with atrial fibrillation (AF), but little is known about the association of AF with the inflammatory serum cytokines after the acute postoperative phase. Thus, we aimed to explore how plasma cytokines concentrations modify during a 3-week cardiac rehabilitation after heart surgery, comparing patients who developed postoperative AF (POAF) and those with permanent AF with patients free from AF (NoAF group). We enrolled 100 consecutive patients and 40 healthy volunteers as a control group. At the beginning of cardiac rehabilitation, 11 days after surgery, serum levels of MPO, PTX3, ADAM17, sST2, IL-25, and IL-33 were dramatically higher, whereas TNFα and IL-37 levels were much lower in NoAF, POAF, and permanent AF patients than in the healthy volunteers. After rehabilitation, most of the cytokines changed tending towards normalization. POAF patients (35% of the total) had higher body mass index and abdominal adiposity than NoAF patients, but similar general characteristics and risk factors for POAF. However, ADAM-17 and IL-25 were always lower in POAF than in NoAF patients, suggesting a protective role of IL-25 and ADAM 17 against POAF occurrence. This finding could impact on therapeutic strategies focusing on the postoperative prophylactic antiarrhythmic interventions.

## Introduction

Cardiac surgery is frequently complicated by postoperative atrial fibrillation (POAF), which is associated with increased morbidity and costs^1,2^. POAF occurs in a third of patients undergoing cardiac bypass surgery^3^ and up to 40% of patients undergoing valvular surgery^4^, usually within the first three days after the intervention. Post-operative atrial arrhythmias can also occur after non-cardiac surgery, especially after thoracic and large abdominal surgery, but with lower incidence^5^.

Previous studies have demonstrated that inflammation is closely related to the pathogenesis of atrial fibrillation (AF)^6^ and there is strong evidence supporting an association between inflammation and AF. The inflammatory cardiac diseases, such as myocarditis, are known to be associated with an increased incidence of arrhythmia, including AF^7^. Cardiac surgery is an acute stressful event generating a chain of inflammatory reactions, as a consequence of the aortic clamp, the organ reperfusion injury during cardiopulmonary by-pass for extracorporeal circulation and the surgical injury itself. The underlying inflammatory pathway involves leukocyte activation^8^. The inflammatory cascade after the surgery, represented by many circulating cytokines, may play a prominent role in initiating POAF. In fact, the cytokines, a network of intracellular proteins produced by lymphocytes, monocytes, and macrophages in response to inflammatory stimuli, have been assessed as potential mediators in the occurrence of AF^9^.

While a few studies evaluated the concentrations of biohumoral markers of inflammation perioperatively, very little is known about the serum levels of cytokines after the acute post-operative phase, i.e. some days after surgery when the patients enter a cardiac rehabilitation program. Furthermore, to the best of our knowledge, no data are available on the patients’ inflammatory profile at the end of rehabilitation, particularly in POAF and AF patients. This information is important 1) to evaluate the effect of a standard rehabilitation period on the patients’ inflammatory profile and 2) to understand whether the presence of some cytokines is due to an acute response to the surgical stress or if is more intrinsically related to the inflammatory profile of the patients with POAF or permanent AF.

*Aim of the study*. The main aim of this study is, therefore, to explore the inflammatory profile in patients few days after the heart surgery, when they enter a cardiac rehabilitation program, comparing those who developed POAF complication or were affected by permanent AF with patients who had a postoperative course free from POAF. The secondary aim is to investigate how plasma concentrations of pro-inflammatory and anti-inflammatory mediators are modified during the cardiac rehabilitation period, both in patients with and without POAF.

## Methods

This observational prospective, open-label study was carried out at the Cardiology Rehabilitation Department of Don C. Gnocchi Foundation (Santa Maria Nascente Institute) in Milan, Italy, in accordance with the principles of the Helsinki Declaration and good clinical practice, and in observance of anti-discrimination regulations and standard privacy procedures. The protocol was approved by the Ethics Committee of Don C. Gnocchi Foundation, and all the participants gave written informed consent.

### Participants

We consecutively enrolled 100 adult patients (>18 years) hospitalized in our rehabilitative unit the day of discharge from the heart-surgery unit, after elective coronary artery by-pass grafting, valve replacement or repair and/or ascending aorta surgery with a sternotomy, and able to provide written informed consent. We also enrolled 40 healthy volunteers, among the employees of our hospital and their families, to obtain reference values of cytokines plasma level in a healthy group.

### Rehabilitation period

All the patients attended the same rehabilitation program following the international guidelines^10^ as previously described^11^. It consisted of sub-maximal incremental endurance training and breathing exercises. Briefly, within 24 hours of admission, each patient started a standardized therapy protocol of supervised physical training with five daily sessions of cycling for 3 weeks until the discharge. Each session was performed at 70% maximal heart rate for at least 30 minutes. The sessions duration increased by ten minutes every three days, up to 50 minutes twice a day if this was permitted by the patient’s conditions. Training included limb flexion, extension, and abduction; neck flexion and extension; and trunk flexion, extension, and rotation. The breathing exercises consisted of taking long deep breaths with an inspiratory volume incentivator: the patient was asked to inhale slowly, hold his/her breath for not less than five seconds, and exhale normally.

At admission in our Rehabilitative Unit, a complete blood test was performed on blood samples collected from peripheral veins. A continuous remote electrocardiogram (ECG) monitoring was performed during the first 72 hours. Close clinical surveillance was done during the whole stay in the rehabilitation unit and the ECG was performed in case of symptoms. POAF was defined as any documented episode of AF, lasting more than five minutes or requiring anti-arrhythmic therapy, which occurred after heart surgery, before the admission to our cardiac rehabilitation department or during the in-patient cardiac rehabilitation time.

Patients were examined by transthoracic 2D echocardiographer (Philips Epic 7 C with 3.5 MHz transducer) in the left lateral decubitus position. The left ventricle ejection fraction was calculated as the percentage of changes in left-ventricle volumes between diastole and systole calculated by the biplane Simpson’s rule, in apical 4-chamber view. The left atrium (LA) size was measured from standard apical 4-chamber views at ventricular end-systole, just before mitral valve opening. LA borders, consisting in the walls of the LA excluding pulmonary veins and LA appendage, were traced using planimetry. The LA area was calculated using the single plane method and the LA index expressed as the ratio between LA area and body-surface area, this latter estimated from the patient’s height and weight by the Gehan and George formula^12^.

### Enzyme-linked immunosorbent assay (ELISA)

In all the 100 patients and 40 controls, serum was collected in vacutainer tubes containing serum separator (Becton Dickinson and Co.). After 40 min at room temperature, samples were centrifuged at 3,000 rpm for 10 min to separate sera. Serum was used immediately or stored at −80 °C. Tumor necrosis factor alpha (TNFα), Myeloperoxidase (MPO), ADAM-17, sST2, Transforming growth factor beta1 (TGF-β1), Interleukin 1beta (IL -1β), IL-25, IL-18, IL-10, IL-8, IL-13, IL33, IL-37, Pentraxin-related protein 3 (PTX3) and Endothelin concentrations were analyzed in serum by sandwich immunoassays according to the manufacturer’s recommendations (Quantikine Immunoassay; R&D Systems, Minneapolis, MN, USA or LSBio, Life Span Bioscience, Seattle WA). A plate reader (Sunrise, Tecan, Mannedorf, Switzerland) was used and optical densities were determined at 450/620 nm. All samples were performed in duplicates. In the patients’ group, the cytokines serum levels were measured both at the time of admission (T0) and at the time of discharge (T1) from our rehabilitation unit.

### Statistics

Patients were divided into four groups: patients who experienced postoperative AF (POAF group), patients with permanent AF (AF group), patients with a previously implanted pacemaker (PM group), and all the other patients (NoAF group). Therefore, patients in the NoAF group did not experience AF neither in the heart-surgery nor in the rehabilitative units.

To characterize the inflammatory status of each patients group at the admission of rehabilitation, the cytokines levels measured at T0 were compared with those measured in the reference group of healthy volunteers by the Mann-Whitney U test. Then, general characteristics of the NoAF group were compared with those of each of the other groups by the Mann-Whitney U test for ordinal variables and by Fisher’s exact test for categorical variables, with p = 0.05 the threshold for statistical significance.

To describe the impact of postoperative and permanent AF and the effect of the rehabilitation period on the inflammatory status, we compared the measures at admission and end of the rehabilitation by groups. For this comparison, first, the distributions of cytokines levels measured at T0 and T1 in each patients’ group were tested by the Shapiro Wilk normality test, possibly after a Box-Cox transformation to obtain distributions closer to the Gaussian distribution. If the hypothesis of Normality was not rejected (p > 0.05), cytokines levels were tested by mixed ANOVA with within-subjects factor the time of measure and between-subjects factor the patients’ group. If one of the factors or their interaction were significant, a post-hoc analysis was performed by the Fisher’s Least Significant Difference test. On the other hand, if the hypothesis of Normality was rejected even after Box-Cox transformations, differences between T0 and T1 in cytokines levels were tested in each group by the Wilcoxon Matched Pairs test and differences between the NoAF group and each of the other groups were tested by the Mann Whitney U test, at T0 and T1 separately. Since most of cytokines distributions are markedly asymmetric, the distributions central tendency was expressed by the median value, and the distribution dispersion around the median by the inter-quartile range or by the median of the absolute deviations from the median (MAD).

## Results

The patients were admitted to our Rehabilitative Unit 10.8 (5.7) days after surgery, mean (SD), and the T0-T1 observation period, i.e. from admission to discharge from the Rehabilitative Unit, lasted 21.7 (6.2) days. The surgery required extracorporeal circulation in 76% of the patients.

### Patients composition

**Figure 1:**
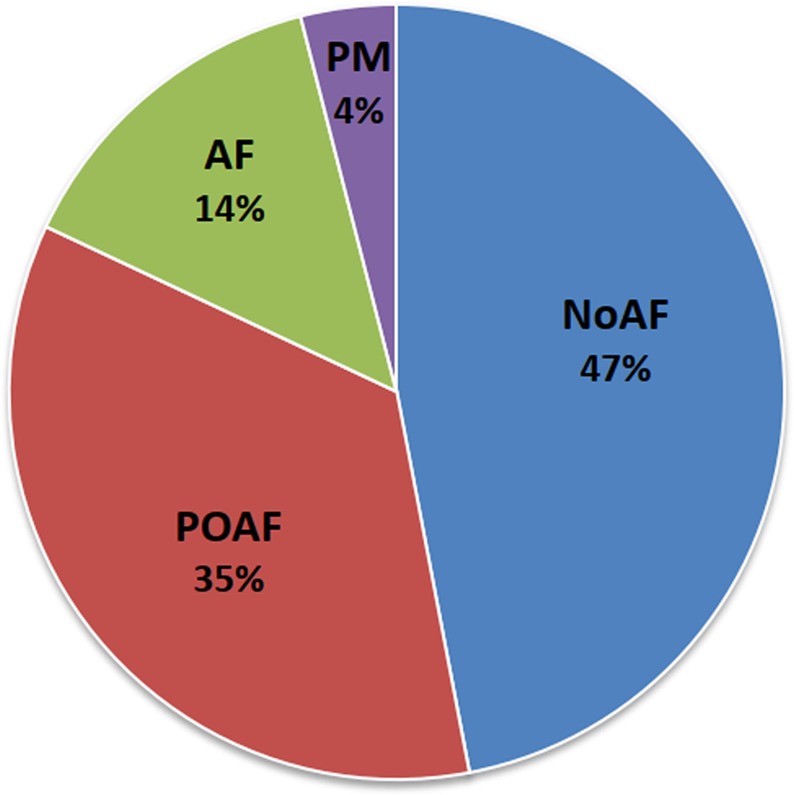
Frequency distribution of the four groups enrolled (N = 100). PM = patients with a previously implanted pacemaker; AF = patients with permanent atrial fibrillation; POAF = patients with postoperative atrial fibrillation; NoAF=patients who did not experience atrial fibrillation.

Over the group of 100 patients, 35 experienced POAF (POAF group), most of them (34 patients) during the hospitalization in the heart-surgery department and one patient on day 1 of admission to our rehabilitation unit. In the POAF group, 15 patients had one or more relapses during the observation time. Furthermore, other 14 patients were affected by permanent AF (AF group) and 4 patients had a previously implanted pacemaker (PM group). The remaining 47 patients did not experience atrial fibrillation, neither in the heart-surgery department nor in the cardiac rehabilitation department (NoAF group). Figure 1 shows the frequency distribution of the four groups. Due to its low sample size (n = 4), we excluded the PM group from the successive statistical comparisons (a descriptive statistics of these patients is reported in Table S1 of the supplemental information). After the exclusion of the PM group, the patients’ age was 71 (10.6) yrs, the body mass index was 25.1 (4.2) kg/m^2^, the prevalence of males was 61%.

Table 1 compares cytokines levels in the NoAF, POAF and AF groups with those of the healthy volunteers, at the rehabilitation program admission. Healthy volunteers had a similar prevalence of the male sex (58%, p = 0.85 after Fisher exact test) and a slightly lower age of 66.2 (10.6) yrs (p = 0.02) in comparison to the whole group of patients. The table shows dramatically lower levels of TNFα and IL-37 and much higher levels of MPO, PTX3, ADAM17, sST2, IL-25, and IL-33 in all the patients’ groups compared to the healthy controls. NOAF patients had significantly higher serum levels of IL-6 and IL-10 than the healthy controls and the same trend also characterized the POAF and AF groups. By contrast, IL-18 levels were markedly greater in the NOAF group only and IL-13 levels in the AF group only in comparison to controls.

**Table 1:** Cytokines serum concentration levels: comparison between healthy controls and each patients’ group at T0: median (MAD).

	Controls (N = 40)	NoAF (N = 47)	p	POAF (N = 35)	p	AF (N = 14)	p
*IL-1*β *(pg/mL)*	1.0 (0.5)	1.2 (0.3)	0.11	1.3 (4.0)	0.16	1.3 (4.5)	0.33
*IL-6 (pg/mL)*	8.2 (4.6)	21.1 (11) *	0.03	20.3 (23.9) °	0.06	24.5 (47.2) °	0.10
*TNFα (pg/mL)*	71.9 (50.5)	6.9 (5.6) *	10^−8^	7.3 (16.5) *	10^−9^	7.4 (8.9) *	10^−5^
*MPO (ng/mL)*	10.8 (0.5)	459 (168) *	10^−9^	477 (307) *	10^−9^	432 (221) *	10^−9^
*PTX3 (pg/mL)*	95.1 (25.5)	5775 (4438) *	10^−9^	3103 (18686) *	10^−9^	3189 (9938) *	10^−9^
*ADAM17 (pg/mL)*	14.9 (5.7)	59.2 (25.3) *	10^−9^	51.5 (40.1) *	10^−9^	60 (31.6) *	10^−5^
*sST2 (ng/mL)*	0.85 (0.31)	22.2 (15.5) *	10^−9^	34 (34.3) *	10^−9^	28.6 (64.2) *	10^−9^
*TGF-*β*1 (ng/mL)*	29.7 (10.1)	29.2 (5.8)	0.40	32.7 (15.4)	0.32	22.7 (14.8)	0.16
*IL-25 (pg/mL)*	0.96 (0.57)	3.6 (1.1) *	10^−6^	3.2 (1.5) *	10^−4^	3.5 (1.7) *	0.003
*IL-18 (pg/mL)*	276.3 (85.2)	378.3 (146) *	0.004	297 (349)	0.22	346 (236) °	0.09
*IL-10 (pg/mL)*	18.9 (6.2)	25.5 (5.6) *	0.01	24.8 (5.7) °	0.08	26.2 (7.7) °	0.08
*IL-8 (pg/mL)*	36.6 (19.8)	27.2 (10.1)	0.49	23.4 (27.3) °	0.07	26.7 (21.7)	0.30
*IL-13 (pg/mL)*	198 (80)	224 (140)	0.69	269 (320)	0.18	388 (680) *	0.008
*IL-33 (pg/mL)*	4.6 (1.1)	10.3 (5.3) *	10^−7^	8.5 (9) *	10^−6^	10.7 (10.3) *	10^−4^
*IL-37 (pg/mL)*	2751 (2247)	467 (455) *	10^−5^	172 (842) *	10^−9^	318 (2075) *	10^−3^

p is the statistical significance of the difference vs. Controls after Mann Whitney U test; significant values (p ≤ 0.05) are indicated by *, trends (0.05 < p ≤ 10) by °; MAD = Median Absolute Deviation.

Table 2 compares the general characteristics of AF and POAF groups with those of the NoAF group. NoAF and POAF patients had similar age, gender composition, left-ventricle ejection fraction, prevalence of a history of hypertension and diabetes; also the prevalence of metabolic syndrome did not differ significantly. By contrast, the prevalence of insulin resistance was substantially greater in the POAF than in the NoAF group and POAF patients also had significantly greater waist circumference, body mass index and surface area, as well as higher HDL values. The AF patients were older than NoAF patients. They also had significantly greater LA size and LA index, and lower Triacylglycerol and Ferritin values than NoAF patients.

**Table 2:** General characteristics of NoAF, POAF and AF groups as percentage or median (MAD), with the p-value of the statistical significance vs. NoAF.

	NoAF (N = 47)	POAF (N = 35)	p	AF (N = 14)	p
Time after Surgery (days)	8 (1)	9 (2)	0.29	8 (1)	0.30
Extracorporeal Circulation (%)	78.7%	82.9%	0.59	78.6%	>0.99
age (yr)	71 (6)	71 (5)	0.36	77.5 (6.5) *	0.009
Male gender (%)	66%	60%	0.65	42.9%	0.13
Body Mass Index (kg/m^2^)	24.1 (2.6)	25.8 (2.2) *	0.02	26.4 (2.4) °	0.055
Body Surface Area (m^2^)	1.74 (0.15)	1.85 (0.13) *	0.04	1.87 (0.15) °	0.098
Waist (cm)	93 (7)	99 (9) *	0.048	100 (9.5)	0.19
Weight/Height Ratio	55.8 (4.5)	58.9 (4.0) °	0.06	61.2 (5.6)	0.13
Left-ventricle ejection fraction (%)	57 (4)	58 (3)	0.61	50 (10)	0.22
History of Hypertension (%)	70.2%	71.4%	>0.99	64.3%	>0.99
Diabetes Mellitus (%)	23.4%	20.0%	>0.99	21.4%	0.72
Insulin resistance (%)	51.1%	75.8% *	0.04	50.0%	>0.99
Metabolic Syndrome (%)	44.7%	60.0%	0.19	71.4%	0.24
Observation Time (days)	20 (4)	21 (3) *	0.049	21 (2.5)	0.36
LA size (cm^2^)	21 (2)	22 (4)	0.42	31.5 (5.5) *	10^−5^
LA Index (cm^2^/m^2^)	12.3 (1.8)	11.7 (1.5)	0.48	17 (3.5) *	10^−4^
LA Size Classification					
Normal	38.3%	37.1%	0.82	0.0% *	0.006
Mildly dilated	51.1%	54.3%	0.83	42.9%	0.76
Moderately dilated	10.6%	2.9%	0.39	50.0% *	0.002
Severely dilated	0.0%	5.7%	0.43	7.1%	>0.99
Blood Analysis					
Glycaemia (mg/dL)	94 (10)	97 (10)	0.49	100.5 (20)	0.65
Total Cholesterol (mg/dL)	137 (21)	142 (23)	0.61	132 (25)	0.38
HDL Cholesterol (mg/dL)	30 (6)	35 (6) *	0.04	30.5 (11)	0.98
Triacylglycerol (mg/dL)	109 (29)	108 (23)	0.42	84 (20)*	0.001
C-Reactive Protein (mg/dL)	4.5 (2.24)	4.03 (1.89)	0.93	4.73 (2.29)	0.95
Ferritin (ng/mL)	239 (169)	273 (105)	0.92	115 (74) *	0.045
White Blood Cells (n_*_10^3^/µL)	8.39 (2.19)	9.53 (1.47)	0.44	7.48 (1.52)	0.17
Linfocyte (%)	20.4 (3.2)	18.5 (5.3)	0.14	18.5 (5)	0.24
Vitamin D (ng/mL)	11.2 (5.7)	12.8 (4.6)	0.25	12.2 (7.5)	0.71
Hemoglobin (g/dL)	10.8 (1)	10.6 (0.6)	0.39	10.8 (1)	0.51
Total Protein (g/dL)	5.7 (0.4)	5.5 (0.4)	0.12	5.9 (0.3)	0.55
Troponin I (ng/mL)	0.07 (0.04)	0.05 (0.04)	0.28	0.11 (0.1)	0.67
Endothelin (pg/mL)	1.98 (0.64)	2.46 (0.92)	0.25	2.52 (0.78)	0.20

MAD = Median Absolute Deviation; LA = left atrium; Insulin Resistance when HOMA index >2.5 or presence of diabetes; p is the statistical significance of the difference vs. Controls after Mann Whitney U test; significant values (p ≤ 0.05) are indicated by *, trends (0.05 < p ≤ 10) by°.

Table 3 describes drug treatments received by each patients’ group at T0 and T1. Compared to NOAF patients, AF patients were more frequently treated with oral anticoagulants, loop diuretics, and Digoxin; and POAF patients were more frequently treated with Amiodarone.

**Table 3:** Drug therapy by patients’ groups at admission (T0) and discharge (T1) as the percentage of cases, with the p-value of the statistical significance vs. NoAF.

*Drug Therapy*	T0					T1				
	NOAF (N = 47)	POAF (N = 35)	P vs. NOAF	AF (N = 14)	P vs. NOAF	NOAF (N = 47)	POAF (N = 35)	P vs. NOAF	AF (N = 14)	P vs. NOAF
Beta blockers	74.5%	77.1%	>0.99	85.7%	0.49	78.7%	82.9%	0.78	92.9%	0.43
Loop Diuretics	70.2%	82.9%	0.21	100%	0.03	51.1%	62.9%	0.37	85.7%	0.03
ACE Inhibitors	23.4%	31.4%	0.46	50%	0.09	21.3%	42.9%	0.052	42.9%	0.16
AT1-receptor blockers	8.5%	11.4%	0.72	21.4%	0.34	6.4%	8.6%	>0.99	14.3%	0.32
MRA	12.8%	28.6%	0.09	28.6%	0.22	8.5%	8.6%	>0.99	14.3%	0.61
Ca-Channel Blockers	10.6%	17.1%	0.52	14.3%	0.66	14.9%	14.3%	>0.99	14.3%	>0.99
Transdermal Nitrates	4.3%	2.9%	>0.99	0%	>0.99	0%	0%	>0.99	0%	>0.99
Acetylsalicilic Acid	61.7%	60%	>0.99	57.1%	0.77	61.7%	65.7%	0.82	50%	0.54
Other Antiplatelet drug	25.5%	22.9%	>0.99	14.3%	0.49	25.5%	17.1%	0.43	14.3%	0.49
VKA Oral Anticoagulants	36.2%	48.6%	0.36	92.9%	0.0002	38.3%	54.3%	0.18	92.9%	0.005
LMWH	19.2%	37.1%	0.08	50%	0.04	8.5%	17.1%	0.31	14.3%	0.61
Amiodarone	14.9%	74.3%	0.0001	14.3%	>0.99	14.9%	48.6%	0.013	0%	0.19
Cardiac Glycosides^a^	8.5%	0%	0.13	28.6%	0.07	0%	0%	>0.99	28.6%	0.002
Statins	44.7%	48.6%	0.82	50%	0.77	48.9%	51.4%	>0.99	42.9%	0.77
Allopurinol	12.8%	5.7%	0.46	7.1%	>0.99	21.3%	8.6%	0.14	28.6%	0.72
Proton Pump Inhibitors	87.2%	100%	0.04	85.7%	>0.99	85.1%	94.3%	0.29	92.9%	0.67
Tiroxine	4.3%	11.4%	0.39	14.3%	0.22	6.4%	14.3%	0.28	14.3%	0.32
Benzodiazepines	19.2%	8.6%	0.22	0%	0.10	17%	2.9%	0.07	7.1%	0.67
Insulin	14.9%	11.4%	0.75	7.1%	0.67	10.6%	8.6%	>0.99	7.1%	>0.99
Oral Antidiabetics	8.5%	14.3%	0.49	7.1%	>0.99	8.5%	14.3%	0.49	14.3%	0.61
Corticosteroids	12.8%	11.4%	>0.99	7.1%	>0.99	12.8%	0%	0.04	0%	0.32
NSAID	10.6%	20%	0.34	28.6%	0.19	4.3%	5.7%	>0.99	7.1%	0.55
Antibiotics	21.3%	20%	>0.99	21.4%	>0.99	0%	0%	>0.99	0%	>0.99

^a^Digoxin; LMWH = Low-molecular-weight heparin; MRA = mineralocorticoid receptor antagonist; VKA = Vitamin K antagonist; NSAID = Nonsteroidal anti-inflammatory drug.

### Cytokines serum concentrations: differences among groups

Table 4 compares cytokines levels between the beginning and the end of rehabilitation and among the three patients’ groups after mixed ANOVA for all the markers that passed the Normality test. Table 5 compares cytokines levels between T0 and T1 and among groups after nonparametric tests for PTX3 and IL-10, the two markers for which it was not possible to find a Box-Cox transformation that passed the Normality test.

**Table 4:** Cytokines serum concentrations at T0 and T1 by groups: median (MAD), with the p-value of the significance value of the factors.

	Time	NoAF	POAF	AF	p-value		
		(N = 47)	(N = 35)	(N = 14)	Time	Group	Time×Group
IL-1β (pg/mL)	T0	1.19 (0.31)	1.25 (0.34)	1.31 (0.47)	0.08	0.85	0.53
	T1	1.28 (0.42)	1.31 (0.43)	6.6 (5.73)			
IL-6 (pg/mL)	T0	21.1 (11)	20.3 (10.6)	24.5 (16.7)	10^−6^	0.73	0.72
	T1	11.7 (6.4) **	10.1 (5.5) **	17.5 (10.8) °			
TNFα (pg/mL)	T0	6.9 (5.6)	7.3 (6.3)	7.4 (5.9)	0.003	0.98	0.55
	T1	20.5 (19.6) **	15.0 (14.3)	26.3 (25.5) °			
MPO (ng/mL)	T0	459 (168)	477 (148)	432 (111)	0.65	0.58	0.73
	T1	502 (220)	500 (229)	394 (114)			
ADAM17 (pg/mL)	T0	59.2 (25.3)	51.5 (13.1) #	60 (14.6)	0.34	0.039	0.38
	T1	62.4 (29)	52.3 (14.2) ##	52.7 (16.4)			
sST2 (ng/mL)	T0	22.2 (15.5)	34 (20.1)	28.6 (16.9)	0.002	0.62	0.20
	T1	20.1 (19)	18.7 (16.1)	8.0 (7.7) **			
TGFβ−1 (ng/mL)	T0	29.2 (5.8)	32.7 (7.1)	22.7 (7.4) ##	10^−6^	0.002	0.97
	T1	21.7 (7.5) **	22 (3.8) **	16.6 (3.6) **,##			
IL-25 (pg/mL)	T0	3.62 (1.08)	3.16 (0.69) #	3.50 (0.85)	0.008	0.035	0.28
	T1	2.93 (1.0) **	2.54 (0.62) **,#	3.50 (0.85)			
IL-18 (pg/mL)	T0	378 (146)	297 (117)	346 (104)	0.73	0.63	0.09
	T1	356 (145)	355 (126)	358 (99)			
IL-8 (pg/mL)	T0	27.2 (10.1)	23.4 (7)	26.7 (9.2)	0.84	0.055	0.58
	T1	32.7 (16.1)	21.2 (7)	23.5 (6.3)			
IL-13 (pg/mL)	T0	224 (140)	269 (160)	388 (194) #	0.016	0.64	0.03
	T1	404 (210) **	381 (133)	323 (78)			
IL-33 (pg/mL)	T0	10.25 (5.35)	8.46 (2.67)	10.74 (4.78)	0.72	0.67	0.74
	T1	10.96 (5.48)	10.01 (4.01)	8.85 (1.63)			
IL-37 (pg/mL)	T0	467 (455)	172 (160)	318 (303)	10^−4^	0.36	0.78
	T1	489 (477) **	180 (147) **	266 (229)			

Anova after Box-Cox Transformation; p values indicate the statistical significance of factors; for the post-hoc analysis, the symbols °, * and ** indicate significant differences between times at p < 0.10, p < 0.05 and <0.01; the symbols # and ## indicate significant differences vs. NoAF at p < 0.05 and <0.01.

**Table 5:** Cytokines serum concentrations at T0 and T1 by groups: median (MAD).

	Time	NoAF	POAF	AF	p	p
		(N = 47)	(N = 35)	(N = 14)	NoAF vs POAF	NoAF vs AF
PTX3	T0	5775 (4438)	3103 (2017)	3189 (1916)	0.50	0.38
(pg/mL)	T1	9220 (8236)	2389 (1572)	1891 (830)	0.18	0.38
	p T0 vs T1	0.86	0.08	0.43		
IL-10	T0	25.5 (5.6)	24.8 (3.1)	26.2 (4.5)	0.18	0.54
(pg/mL)	T1	11.7 (3.4)	11.1 (2.3)	13.5 (3.6)	0.12	0.72
	p T0 vs T1	10^−4^	10^−5^	0.001		

Differences between times after Wilcoxon Matched Pairs Test, between groups after Mann-Whitney U test.

The group factor was significant for IL-13, TGFβ-1, ADAM17 and IL-25. As to IL-13 and TGFβ-1, the post-hoc analyses revealed significant differences between the AF and the NoAF groups (Table 4). Differences consisted of 1) a lower TGFβ-1 concentration in the AF group, both at T0 and T1; and 2) a higher IL-13 concentration in the AF group at T0. The significant Time×Group interaction for IL-13 means that the rehabilitation period had a significantly different effect among groups.

As to ADAM17 and IL-25, the post-hoc analyses revealed significantly lower concentrations in the POAF than in the NoAF group both at the beginning and the end of rehabilitation, being the Time×Group interactions not significant (Table 4).

### Cytokines serum concentrations: changes after rehabilitation

**Figure 2:**
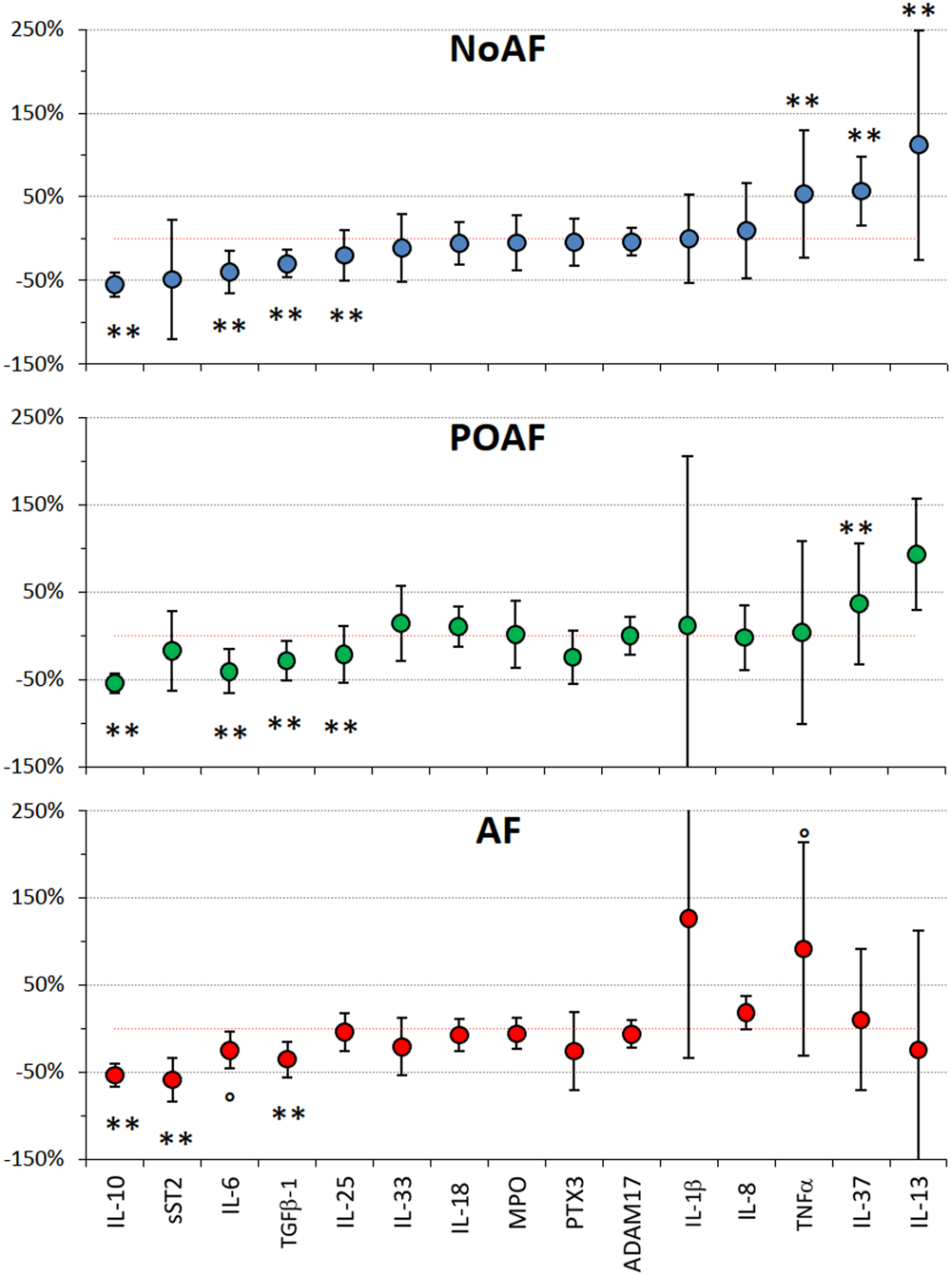
Percent changes in inflammatory markers from admission (T0) to discharge from the rehabilitation program (T1). Values as median ±MAD; the °, * and ** symbols mark changes from T0 to T1 significant at p < 0.10, p < 0.05 and p < 0.01 (see Tables 4 and 5).

Tables 4 and 5 also show that timing is a significant factor for IL-6, TNFα, sST2, TGFβ-1, IL-25, IL-37, and IL-10, which means a significant effect of the rehabilitation period in at least one of the patients’ groups. Figure 2 summarizes the effects of time in each group showing percent changes in the cytokines concentrations from T0 to T1. IL-10 and TGFβ-1 serum concentrations decreased in all the groups; IL-25 and IL-6 decreased in the NoAF and POAF groups only, and sST2 in the AF group only (at T0, serum concentrations of these cytokines but not TGFβ-1 were greater in patients than in healthy controls, see Table 1). On the other hand, the rehabilitation period substantially increased the TNFα concentrations in the NoAF and AF groups and the IL-37 concentration in the NoAF and POAF groups (at T0, the serum concentrations of TNFα and IL-37 were lower in patients than in healthy controls, see Table 1). Rehabilitation also increased the IL-13 concentration in the NoAF group.

## Discussion

Main results of our study are that 1) serum concentrations of different cytokines are consistently altered in patients at admission to the cardiac rehabilitation even 10 days after surgery, compared to healthy controls; 2) the cardiac rehabilitation has beneficial effects on some inflammatory biomarkers; 3) some cytokines concentrations differ between NoAF and POAF groups up to the end of rehabilitation, one month after surgery. In particular, the following points deserve to be discussed in detail.

### General characteristics of NoAF, POAF and AF groups

We found a high incidence of POAF after surgery, as previously reported^2–4^. In most cases, POAF occurred a few days after surgery, as expected^13^, with several relapses during rehabilitation. Among the main risk factors for POAF^14^, some trials indicated older age, left atrial anatomy and left ventricular diastolic dysfunction^3^. However, our POAF and NoAF groups did not differ in terms of postoperative cardiac output, cardiac remodeling, changes in telediastolic and atrial pressure, sympathetic activation or withdrawal of β-sympatholytic drugs, age and most of the general characteristics (Table 2). Therefore, we can exclude preexisting left atrial remodeling or the older age as facilitating factors for POAF in our patients. The POAF group did not show a higher prevalence of metabolic syndrome or diabetes, both reported as risk factors for POAF^15^, but they had a higher HOMA Index, suggesting a role of insulin resistance and glucose intolerance in the genesis of POAF^16^. Their greater abdominal adiposity agrees with the association between waist circumference and the risk of AF^17^. By contrast, AF patients were older than NoAF patients, with bigger left atria. Their lower levels of triacylglycerol and ferritin are likely due to their differences in general characteristics.

Previous studies on circulating biochemical markers and POAF have not been translated into diagnostic or therapeutic indications^18^. In our study, neither the marker of myocardial damage, troponin I (a cardiac protein that controls the calcium-mediated interaction between actin and myosin), nor inflammatory markers as C-reactive protein, ferritin, or endothelin (a vasoconstrictor promoting inflammation correlated with the left atrium size in patients operated for mitral valve disease^19^) differed between NoAF and POAF groups (Table 2), despite evidence supporting a role of inflammation in POAF^20^. This may suggest that the more common proteins used to assess the acute inflammation phase are unlikely to predict the risk of POAF in clinical practice.

### Cytokines serum concentrations at the admission of cardiac rehabilitation

Most cytokines concentrations differed between patients and controls at T0 (Table 1). IL-6 was higher and TNFα lower in patients compared to controls. IL-6 is the primary stimulator of the acute-phase proteins produced by cardiovascular components like endothelial cells, vascular smooth muscle cells, and ischemic cardiomyocytes^21^. It has been shown that the IL-6 serum concentration increases dramatically in the first couple of days after cardiac surgery and then quickly decreases reaching a level similar to what we observed in our patients at T0 six days after surgery^22^. IL-6 stimulates the synthesis of C-reactive protein and fibrinogen and counter-regulates IL-1β and TNFα in humans^23^, thus explaining the much lower serum concentration of TNFα in our patients at T0 than in healthy controls. Our measure of TNFα at T0 was greater than previously reported in patients before coronary artery bypass grafting and slightly greater than measured in the same patients two days after surgery^24^, suggesting a recovery trend of TNFα already at T0.

PTX-3 and sST2, dramatically greater in all the patients’ groups, are associated with cardiovascular damage. PTX-3 is a pleiotropic marker of vascular inflammation in vascular endothelial cells and monocytes in response to proinflammatory cytokines. It predicts cardiovascular morbidity and mortality in unstable angina^25^ and short-term recovery after heart surgery^26^. The member of the interleukin-1 receptor family sST2 is a marker of cardiac remodeling: high levels are associated with fibrosis and hypertrophy in heart failure or coronary heart disease^27^.

IL-33 is much greater and IL-37 much lower in all the patients’ groups at T0 than in controls. IL-37 inhibits the expression of pro-inflammatory cytokines^28^ and in mice exhibited significant improvements in the atherosclerotic burden^29^. Recently it has been suggested a possible role of IL-37 in human sera as a therapeutic agent or biomarker for the diagnosis of cardiovascular diseases^30^. On the other hand, IL-33, secreted by endothelial cells, has pro-inflammatory characteristics and may translate myocardial pressure overload into a selective systemic inflammatory response^31^. IL-33 can activate an acute inflammation by its ligation with ST2; in the late inflammatory phase, it contributes to dampening the inflammatory response stimulating anti-inflammatory monocytes^32^. Further pieces of evidence indicate a protective role of IL-33 in diabetes-related heart complications^33^ and in the development of atherosclerosis^34^.

IL-10, greater in patients at T0 than in controls, promotes the immune responses and the integrity of tissue epithelial layers facilitating the tissue-healing process after infection or inflammation^35^.

IL-18, a member of the IL-1 family, induces inflammation, has broad immunomodulatory properties and plays a critical role in host defense against some infections^36^. Its blockade has atheroprotective effects in murine models^37^. Its higher levels in NOAF patients at T0 than in healthy controls appear a novel finding of our study.

Serum concentrations of IL-1β, IL-8 and TGFβ-1 were similar in patients and controls, likely because eleven days after surgery were sufficient to normalize their levels.

### Changes in cytokines serum concentrations at the end of cardiac rehabilitation

Some cytokines decreased along the rehabilitation time (Table 4). One is IL-6, greater in patients at T0 than in controls. Therefore, the decrease of IL-6 observed acutely a few days after surgery^22^ continues further during the rehabilitation period. The IL-6 decrease could have promoted the parallel increase of TNFα from T0 to T1, given the capability of IL-6 to inhibit the production of TNFα^23^. Interestingly, the TNFα increase during rehabilitation appears blunted in the POAF group compared to NoAF or AF patients (Table 4 and Fig. 2). This could be due to the more frequent use of Amiodarone in the POAF group at T1 because it was shown to attenuate the production of TNFα^38^.

Also, IL-10 and TGF- β1 decreased during the rehabilitation period in all the patients’ groups. Since IL-10 facilitates the tissue-healing process, its decrease during the rehabilitation period should be considered a favorable result. Growth factors are involved in the overall inflammatory response and probably TGF- β1 is also associated with the healing of surgical injury during rehabilitation. Furthermore, sST2 decreased in the AF group, or because of the improvement of heart compensation along with the rehabilitation, or because of a drug effect: beta-blockers affect sST2 levels^39^ and some interferences of therapies are possible.

By contrast, IL-37, which exerts an anti-inflammatory effect in inflammatory diseases^36^ increased during the rehabilitation period in the NoAF and POAF groups. Since IL-37 was much lower in patients at T0 than in controls, its increase should be considered a beneficial effect of the rehabilitation period.

The serum concentrations of MPO, PTX3, ADAM17, IL-18, and IL-33, much greater in patients at T0 than in controls, did not change over time, suggesting that they may undergo to persistent changes in cardiac patients and that a standard cardiorespiratory rehabilitation program is insufficient to reduce their levels.

### Differences in cytokines serum concentrations between POAF and NoAF groups

Some cytokines appeared in the scientific literature as promising markers for identifying patients at risk for the POAF development^40^. In this regard, we found a lower plasma concentration of the metalloproteinase ADAM-17^41^ in POAF patients. The difference does not depend on the time of observation, appearing both at T0 and T1, and suggests that it is not secondary to the onset of POAF. These findings may indicate a possible protective role of ADAM-17 against POAF occurrence. Even if it is far to be fully clarified which components of the inflammatory response are protective or harmful, ADAM 17, a cytokine associated with inflammation and worse prognosis in heart diseases^42^, belongs to the adamalysin protein family, which is known to determine a release of adhesion molecules^43,44^. The favorable action of ADAM-17 could be exerted through the release of adhesion molecules that enable macrophages to migrate into the inflamed atrial tissue, preventing their amplification of local inflammation, a mechanism of action that likely might be critical in determining the onset of POAF. There is also evidence of a possible role of ADAM-17 in the shedding of the junctional adhesion molecule 1, a sort of zipper between endothelial cells^45^. In transgenic mice the inhibition of ADAM-17 prevented the dilation of the left ventricle, probably preserving TNFα on myocyte surfaces^46^ suggesting a role of ADAM-17 in cardiac remodeling^47^. On the other hand, lower ADAM-17 levels may have a clinical linkage with insulin resistance in POAF patients. In fact, an ADAM-17 inhibitor restored insulin sensitivity to normal in insulin-resistant non-obese diabetic rats^48^. The influence of ADAM-17 on glucose homeostasis seems mediated by TNFα inhibition.

We also found significantly lower plasma levels of IL-25 in POAF patients and, also in this case, the difference persisted from T0 to T1. IL-25 plays a role in some inflammatory diseases, coordinating intercellular processes between many cell types and being involved in the immune response to foreign pathogens. In particular, it has a potent reciprocal interaction with other members of the IL-17 family, such as IL-17A, and blocking IL-25 results in an IL-17A increase, as demonstrated in a clinical contest^49^. IL-17A is involved in inflammatory tissue destruction and vascular disease, favoring the accumulation of leukocytes, and immune cells into the vessels wall, both in acute vasculitis and in chronic forms of vascular disease such as atherosclerosis^50^. We might speculate that the elevated IL-25 circulating levels exert a favorable effect in counteracting the strong inflammatory response that IL 17 A induces on vessels and ultimately within the atrial tissue: this could result in a protective effect against POAF.

**Figure 3:**
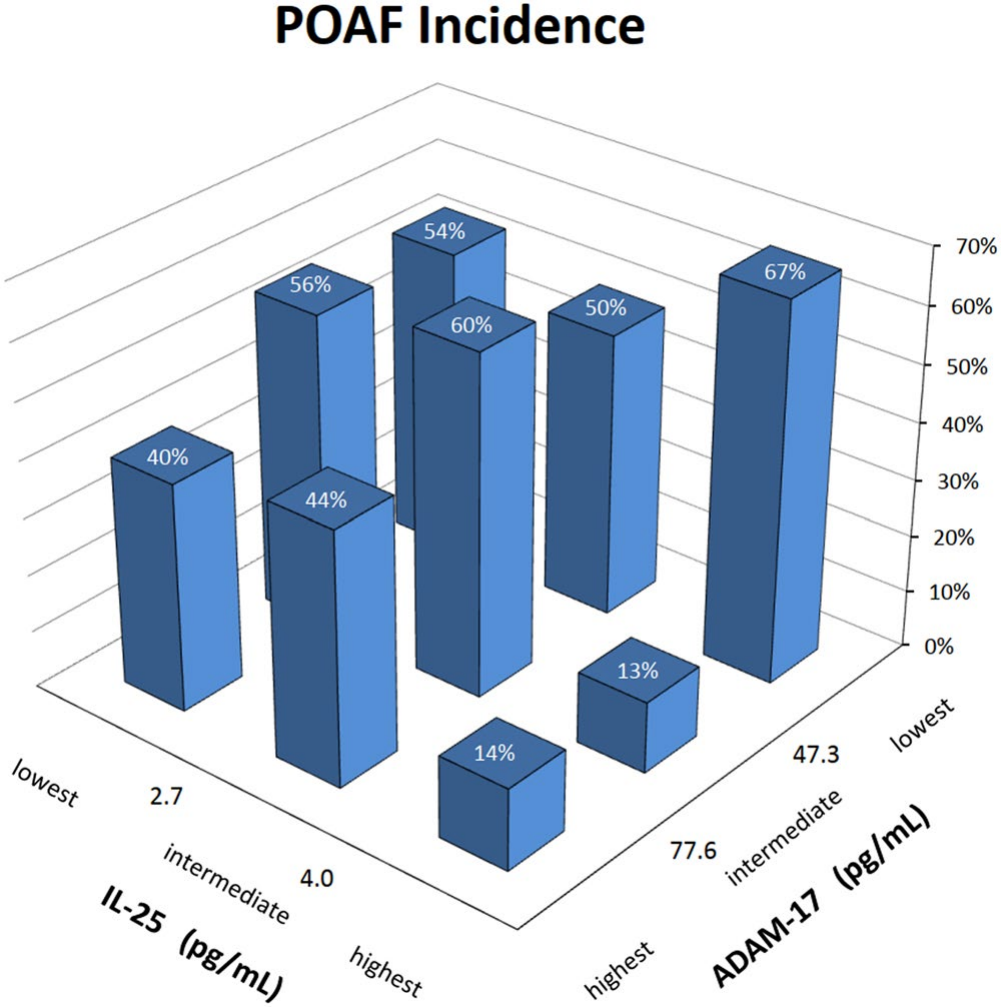
POAF incidence by tertiles of IL-25 and ADAM-17. Each column represents the percentage of cases who developed POAF after surgery, separately from the lowest to the highest tertile of IL-25 and from the lowest to the highest tertile of ADAM-17 (concentrations assessed as the average between the values measured at T0 and T1). Patients with permanent AF or with implanted pacemaker before surgery are excluded.

The differences in ADAM-17 and IL-25 average values between POAF and NoAF patients may appear modest but, if combined, they might have a remarkable clinical impact. This is illustrated in Fig. 3 that shows the prevalence of POAF in 9 subgroups of NoAF and POAF patients defined by tertiles of IL-25 and ADAM-17. The figure suggests that IL-25 concentrations higher than 4 pg/mL might have a protective effect against POAF which, however, could be completely nullified by a concentration of ADAM-17 below 47.3 pg/mL. Therefore ADAM-17 concentrations lower than 47.3 pg/mL might represent a risk factor for POAF. The figure also suggests that if the cytokines levels fall in the “safe zone”, defined by IL-25 greater than 4 and ADAM-17 greater than 47.3 pg/mL, we may expect only one every 7 or 8 patients to have POAF. By contrast, if ADAM-17 is lower than 47.3, or lower than 77.6 with IL-25 lower than 4 pg/mL, we may expect that the majority of the patients has POAF.

### Limitations

Our study was conducted in a rehabilitation department that receives patients from surgery units of several hospitals for a standardized cardio-respiratory rehabilitation program. This limited the study design as we could not measure the serum cytokines levels before surgery, due to the inability to know which patients would be referred to our unit after surgery. However, to the best of our knowledge, our study provides the first description of the inflammatory profile through several cytokines serum levels in a population of cardiac patients, at the discharge from the cardiac surgery unit, about 11 days after surgery, providing the further changes in the following 21 days of rehabilitation.

### Conclusion

Our results, highlighting that significant lower values of IL-25 and ADAM 17 persist during the whole rehabilitation program in the POAF group, suggest a protective role of these cytokines against the occurrence of POAF. Extensive investigations that include pre-surgery measures are required to confirm the predictive role of these biomarkers. If proven, they could impact future therapeutic strategies, helping clinicians to identify the subjects at higher risk to develop POAF, better focusing the postoperative prophylactic antiarrhythmic interventions.

## Supplementary information

Supplementary Information.

## Data Availability

The datasets generated and analyzed in the current study are not publicly available due to a request from our ethical committee but are available from the corresponding author on reasonable request.
