# Supplementary material for: Inflammatory Cytokines During Cardiac Rehabilitation After Heart Surgery and Their Association to Postoperative Atrial Fibrillation
Source: Sci Rep. 2020 May 25;10:8618. doi: 10.1038/s41598-020-65581-1 (PMC7248057; doi:10.1038/s41598-020-65581-1)
Supplement: Supplementary file 1 — Supplementary Information. [file 41598_2020_65581_MOESM1_ESM.pdf]

Inflammatory Cytokines During Cardiac Rehabilitation After Heart Surgery and Their Association to Postoperative Atrial Fibrillation

Vittorio Racca * MD [a], Anna Torri, MD [a], Paola Grati, MD [a], Claudia Panzarino, MS [a], Ivana Marventano MS [c], Marina Saresella, MS [c], Paolo Castiglioni, PhD [b]

a) IRCCS Fondazione Don Carlo Gnocchi, Milan, Italy, Istituto Santa Maria Nascente, Cardiology Rehabilitation Department

b) IRCCS Fondazione Don Carlo Gnocchi, Milan, Italy

c) IRCCS Fondazione Don Carlo Gnocchi, Milan – Italy, Istituto Santa Maria Nascente, Laboratory of Molecular Medicine and Biotechnology

Supplementary Information: Patients with implanted pacemaker

Four of the consecutively enrolled patients (3 males) had a previously implanted pacemaker. Their age was 73 (12.1) years, their BMI was 27.4 (3.9) kg/m^2, and their left ventricle ejection fraction was 41.8 (10.3) %. This PM group was excluded from all the statistical comparisons with the other groups due to its low sample size. However, this appendix reports the descriptive statistics of the inflammatory markers for the PM group in the following table S1.

Table S1. Cytokines serum concentrations at T0 and T1 in the PM group (N=4): median (MAD)

	T0	T1	p-value
**IL-1β (pg/mL)**	0.89 (0.05)	0.97 (0.03)	0.07
**IL-6 (pg/mL)**	17.4 (7.7)	8.3 (2.6)	0.47
**TNFα (pg/mL)**	0.8 (0.2)	0.8 (0.2)	0.47
**MPO (ng/mL)**	287.7 (27.1)	426.3 (124.8)	>0.99
**ADAM17 (pg/mL)**	63.5 (6.7)	62.4 (12.3)	0.14
**sST2 (ng/mL)**	25.5 (9.1)	20.9 (13.8)	0.47
**TGFβ-1 (ng/mL)**	29.0 (2.2)	21.8 (3.3)	0.27
**IL-25 (pg/mL)**	3.7 (0.6)	2.6 (0.9)	0.72
**IL-18 (pg/mL)**	455 (142.8)	523.9 (202.5)	0.72
**IL-8 (pg/mL)**	27.6 (5.9)	28.2 (3.1)	0.47
**IL-13 (pg/mL)**	407 (199)	527 (168)	0.47
**IL-33 (pg/mL)**	10.4 (2.2)	8.4 (1)	0.72
**IL-37 (pg/mL)**	1756 (1743)	1368 (1264)	0.47
**PTX3 (pg/mL)**	21420 (345)	19525 (2130)	0.07
**IL-10 (pg/mL)**	23.6 (3.1)	16.2 (2.1)	0.14
